# Genome Sequence of *Serratia plymuthica* A153, a Model Rhizobacterium for the Investigation of the Synthesis and Regulation of Haterumalides, Zeamine, and Andrimid

**DOI:** 10.1128/genomeA.00373-16

**Published:** 2016-05-19

**Authors:** Miguel A. Matilla, Alison Drew, Zulema Udaondo, Tino Krell, George P. C. Salmond

**Affiliations:** a Department of Biochemistry, University of Cambridge, Cambridge, United Kingdom; b Department of Environmental Protection, Estación Experimental del Zaidín, Consejo Superior de Investigaciones Científicas, Granada, Spain; c Abengoa Research, Biotechnology Technological Area, Sevilla, Spain

## Abstract

The rhizobacterium *Serratia plymuthica* A153 is a Gram-negative bacterium belonging to the family *Enterobacteriaceae*. Here, we present the genome sequence of this strain, which produces multiple bioactive secondary metabolites, including the halogenated macrolide oocydin A, the polyamino antibiotic zeamine, and the bacterial acetyl-CoA carboxylase inhibitor andrimid.

## GENOME ANNOUNCEMENT

*Serratia plymuthica* strains are widely distributed, and commonly found associated with plant roots (1). They are effective biocontrol agents and plant growth-promoting bacteria, mainly due to their capacity to produce exoenzymes, phytohormones, and various secondary metabolites, coupled with their ability to induce systemic resistance (1, 2).

*Serratia plymuthica* A153 was isolated from the rhizosphere of wheat (3) and synthesizes multiple bioactive secondary metabolites, including several antifungal, antioomycete, and anticancer haterumalides/oocydins (4), the bacterial acetyl-CoA carboxylase inhibitor andrimid (5), the polyamino antibiotic zeamine (6), and the broad spectrum antifungal compound pyrrolnitrin (4). *Serratia plymuthica* A153 was used to identify the biosynthetic cluster encoding synthesis of the haterumalide oocydin A (7). Furthermore, it was used as a model bacterium for the study of the regulation of multiple bioactive non-ribosomal peptides and polyketides (5, 6, 8)—research which was greatly facilitated by the facile genetic tractability of A153 and the isolation of a highly efficient generalized transducing phage, ϕMAM1 (9).

The sequencing of the genomic DNA of *S. plymuthica* A153 was performed at the Department of Biochemistry (University of Cambridge) using 454 DNA pyrosequencing technology on a picotiter plate for a Roche Applied Science Genome Sequencer FLX system. The 454 data were *de novo* assembled using Newbler v2.6. The assembly used 308,585 reads (129 MB of raw data) to give an approximately 22× coverage of the estimated genome size and resulted in a total of 24 contigs larger than 500 bp. The average contig size was 230,980 bp and the largest contig was 1,516,666 bp. The contigs were ordered and oriented based on the whole-genome sequences of the *Serratia plymuthica* strains AS9 (10), AS12 (11), and 4Rx13 (GenBank accession no. CP006250). Traditional Sanger sequencing was used to close the gaps between contigs. The genome was automatically annotated using NCBI Prokaryotic Genomes Annotation Pipeline (PGAP) version 3.0 (http://www.ncbi.nlm.nih.gov/genome/annotation_prok).

The assembled genome of *Serratia plymuthica* A153 consists of 2 large contigs and includes 5,475,375 bp, with an overall G+C content of 55.94%. Automated genome annotation predicted 4,809 protein-coding sequences (CDSs), 30 pseudogenes, 21 rRNA operons, 81 tRNA genes, and 11 noncoding RNAs. In addition to the gene clusters responsible for the biosynthesis of oocydin A, zeamine, and andrimid, antiSMASH (12) predicted 6 additional clusters putatively involved in the synthesis of nonribosomal peptides and polyketides. Genome comparison analyses revealed that the genome of A153 shows high sequence homology with the genomes of the *Serratia plymuthica* strains AS9 (10), AS12 (11), AS13 (13), S13 (14), RVH1 (15), 4Rx13 (GenBank accession no. CP006250), and V4 (16). However, these strains lack several clusters for polyketide and non-ribosomal peptide biosynthesis that are present in A153. The sequencing of the genome of *S. plymuthica* A153 will enable further research on the biosynthesis and regulation of both the known and putatively novel secondary metabolites produced by this strain.

### Nucleotide sequence accession number.

The sequences obtained by this whole-genome shotgun project have been deposited in DDBJ/EMBL/GenBank under the accession number LRQU00000000.
